# Total thoracic herniation of the liver: a case of delayed right-sided diaphragmatic hernia after blunt trauma

**DOI:** 10.1186/s40792-020-00941-7

**Published:** 2020-07-22

**Authors:** Satish Kesavaramanujam, Michael C. Morell, Dhanush Harigovind, Chandana Bhimmanapalli, Sebastiano Cassaro

**Affiliations:** 1 Department of Surgery, Kaweah Delta Health Care District Medical Center, 202 West Willow Avenue, Suite 402, Visalia, California USA; 2 Department of Surgery, University of California, Irvine, California USA

**Keywords:** Diaphragmatic injury, Blunt torso trauma, Diaphragmatic hernia

## Abstract

**Background:**

Traumatic diaphragmatic injuries (TDIs) are relatively uncommon and require surgical repair to prevent or address herniation. Three quarters of TDIs are due to blunt thoraco-abdominal trauma. In blunt TDIs, variable clinical presentations and frequent concurrent life-threatening injuries may hinder early recognition and treatment, leading to diagnostic delays, which may result in technically more challenging repairs. Right-sided blunt TDIs are much less common than left-sided ones, are difficult to visualize on imaging studies, are more frequently associated with other potentially lethal injuries, and tend to present more subtly, so that diagnostic delays are more likely.

**Case presentation:**

We report the diagnosis and elective repair of a large right-sided traumatic diaphragmatic hernia resulting from a distant blunt abdominal injury, describing the techniques used to address the challenges presented by the chronic intrathoracic displacement of the entire liver with the gallbladder, as well as the right side of the colon and part of the duodenum.

**Conclusions:**

Early diagnosis of right-sided TDIs can be especially elusive. The management of delayed diaphragmatic hernias can be challenging, but with meticulous planning and a flexible surgical approach, a repair can be achieved resulting in good recovery and low risk of recurrence.

## Background

Traumatic diaphragmatic rupture is an infrequent clinical entity and an often-missed diagnosis at the time of the inciting event, with as many as two thirds of these injuries being missed at the time of initial trauma [1]. The resulting diaphragmatic hernias may not be detected for years due to their variable anatomical progression and clinical presentation [2].

Post-traumatic right-sided diaphragmatic hernias are much less common than left-sided ones, present more subtly, and are more frequently associated with other life-threatening injuries and a higher mortality rate. The presence of the liver may mask the injury and obscure imaging studies. Right-sided diaphragmatic ruptures are therefore more likely to be missed at the time of initial trauma and to present at a later time with symptoms due to herniation of abdominal organs into the thoracic cavity [3].

We present the case of a 35-year-old male with a remote history of a motor vehicle accident who presented with muted symptomatology and was found to have a massive right diaphragmatic hernia, with the entire liver, gallbladder, part of the colon, stomach, omentum, and part of the duodenum displaced into the thorax.

## Case presentation

A 35-year-old male presented to the emergency department (ED) with intermittent shortness of breath and right upper quadrant pain associated with significant bloating following meals. He reported multiple previous ED visits for the same symptoms. The patient’s other significant medical conditions included asthma, obesity, and seropositive rheumatoid arthritis being treated with disease-modifying anti-rheumatic drugs and systemic steroids.

The patient gave a history of a motor vehicle crash several years ago. The patient was the front seat passenger of a vehicle which was struck on his side by an incoming vehicle. The patient reported that a computed tomography (CT) scan done at the time revealed only several broken ribs, but he recalled no mention of a diaphragmatic injury.

**Fig. 1:**
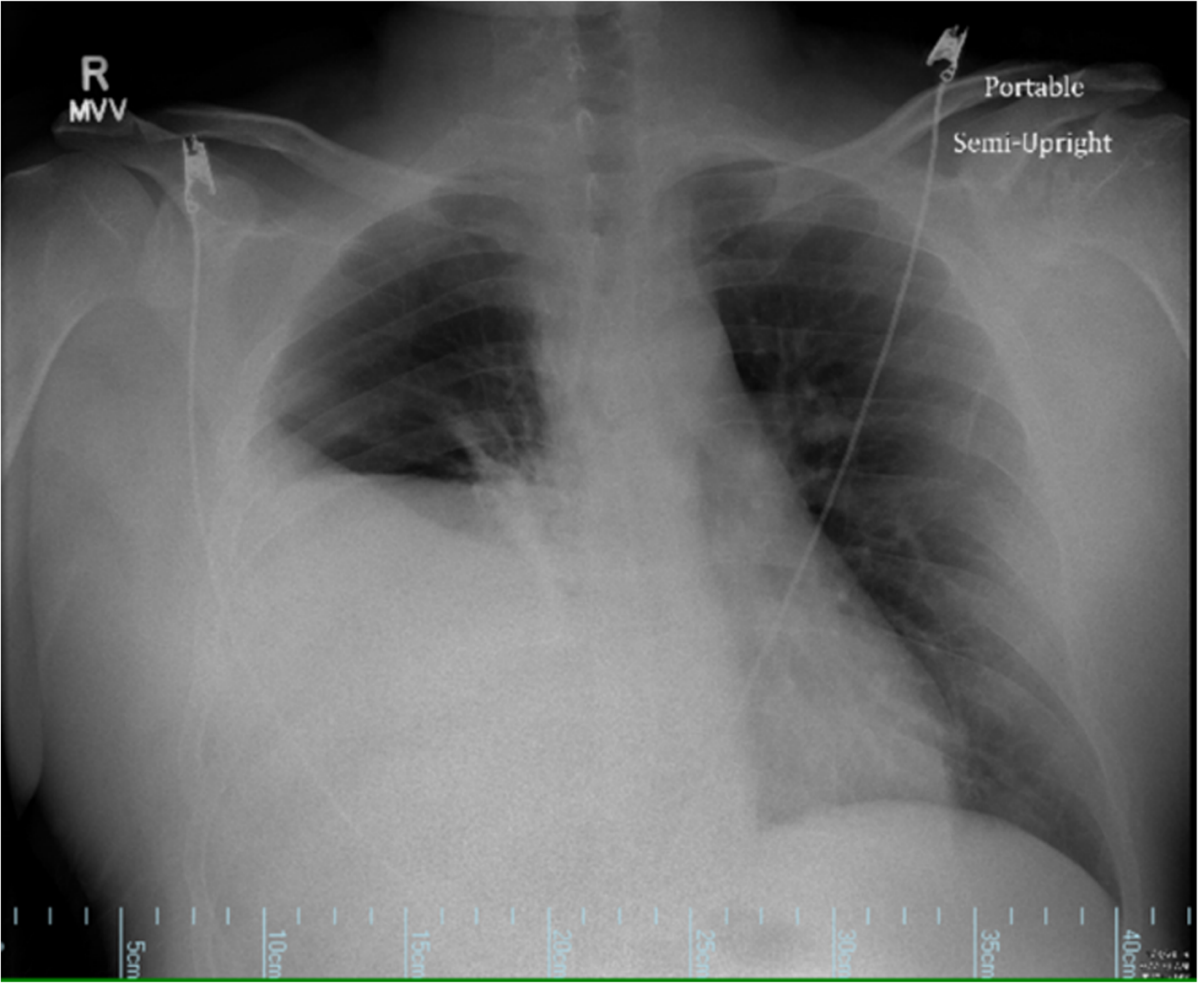
Pre-op chest radiograph showing nonspecific elevation of the right hemidiaphragm and left mediastinal shift

**Fig. 2:**
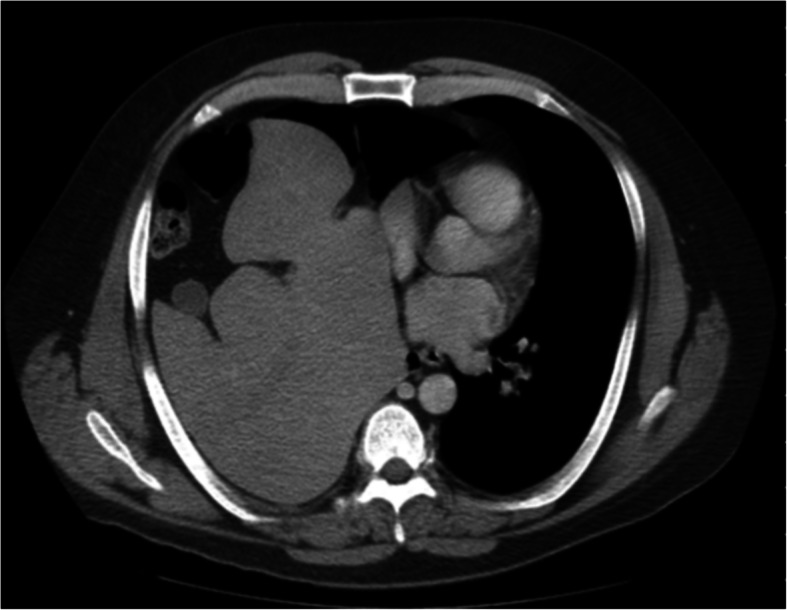
Axial chest CT showing the liver, gallbladder, and loop of bowel in the right thoracic cavity

**Fig. 3:**
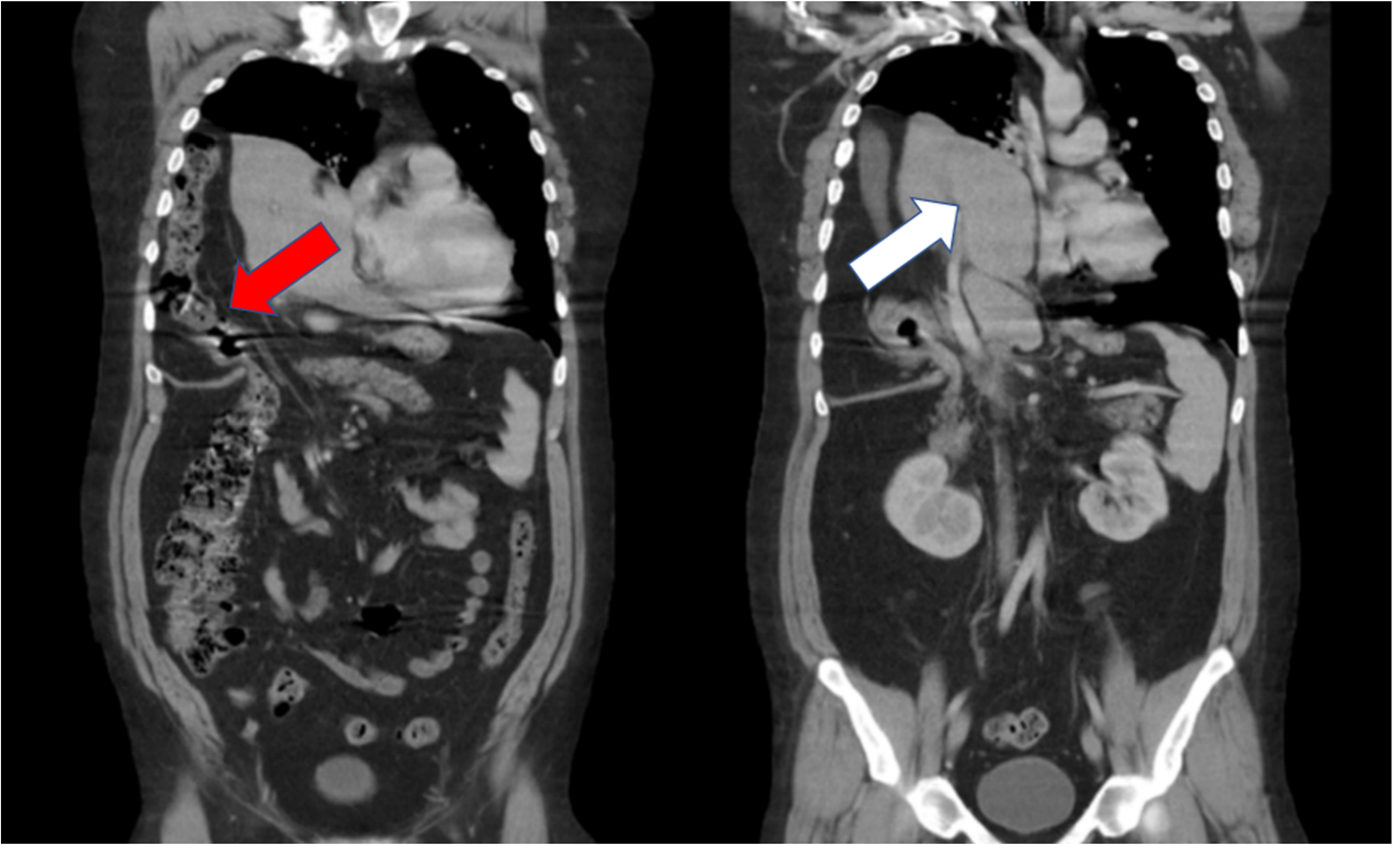
Coronal chest/abdominal/pelvis CT showing massive thoracic displacement of intraabdominal organs. In the left image, a loop of the colon can clearly be seen entering the thoracic cavity through the defect (red arrow). In the right image, the entire mass of the liver can be seen in the thoracic cavity with the hepatic vasculature extending across the diaphragmatic defect (white arrow)

Chest radiograph showed a significant elevation of the right hemidiaphragm and mild resultant leftward shift of the mediastinum (Fig. 1). A CT of chest, abdomen, and pelvis showed a 15- by 10-cm right diaphragmatic defect resulting in the intrathoracic herniation of the entire liver, the gallbladder, the hepatic flexure of the colon, and the descending duodenum (Figs. 2 and 3). There was no evidence of bowel obstruction or gallbladder pathology.

At the time of the initial diagnosis of the diaphragmatic hernia, the patient was being treated with 10 mg of oral prednisone twice a day for rheumatoid arthritis. Given his stable clinical picture, lack of obstructive symptoms, and the high dose of scheduled systemic steroids, the patient was discharged home and prepared for surgery. After weaning him down to 2 mg of oral prednisone twice daily, the patient underwent an elective surgical repair.

**Fig. 4:**
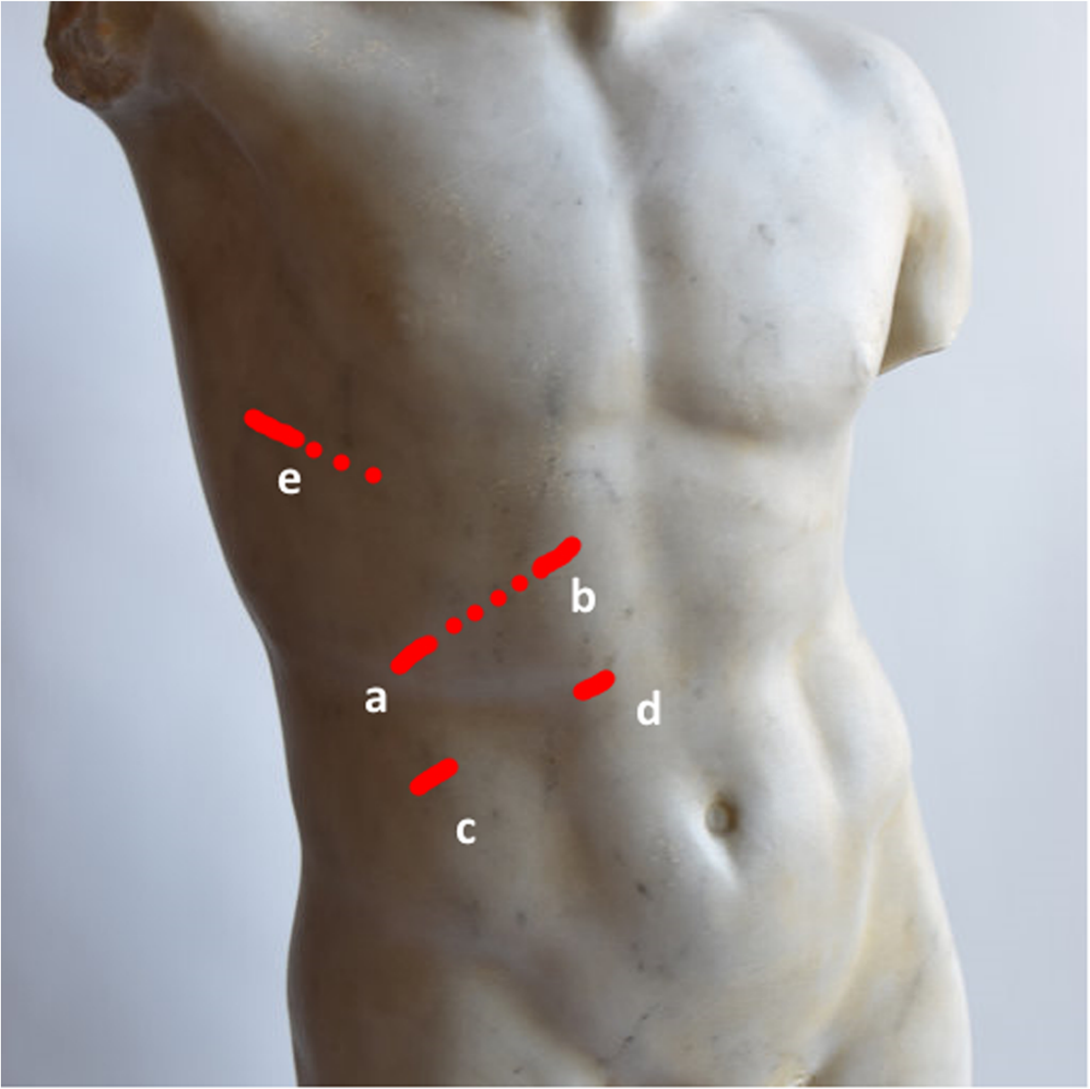
Diagram illustrating port placement sites referenced in the text. The dotted lines represent the extension of the port incisions used to complete the procedure

**Fig. 5:**
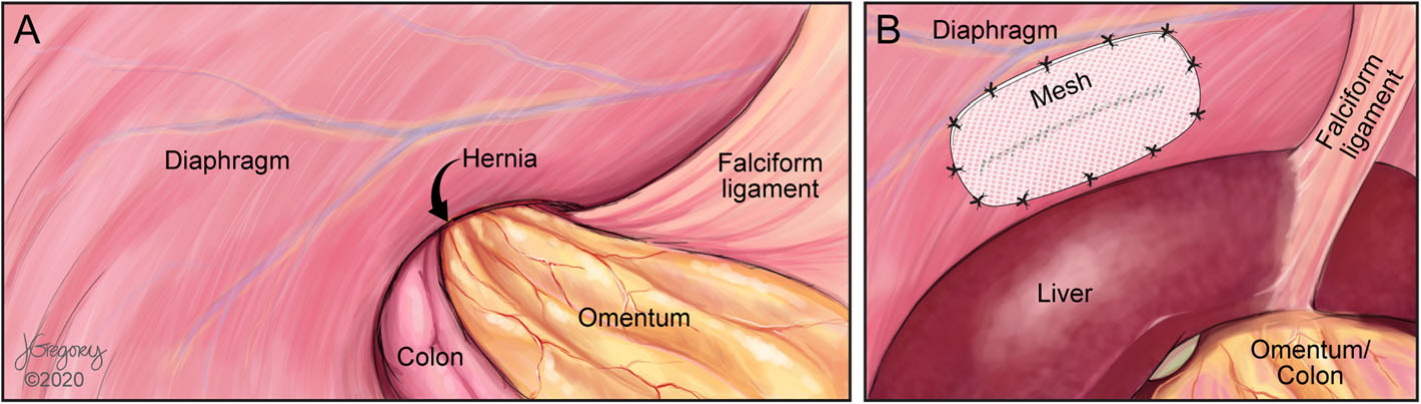
**a** Illustration of the initial laparoscopy findings, with the herniation of the abdominal organs through the right diaphragm. Note the absence of the liver in its normal anatomic position. **b** Illustration of the laparoscopy view at the completion of the procedure with the repaired diaphragm and the Covidien ProGrip® mesh overlay

Given the complexity of the altered anatomy, the procedure was carried out using a combined hand-assisted thoracoscopic-laparoscopic approach. Initially, a 5-mm Covidien Visiport Plus (Medtronic, Minneapolis, MN) optical trocar was placed under vision in the right upper quadrant (Fig. 4a), and the abdomen was insufflated to 15 mm Hg. Three additional 5-mm ports were placed in the right upper quadrant (Fig. 4b–d). On laparoscopy, a large hernia defect was apparent in the right hemidiaphragm (Fig. 5a). The liver was absent from its anatomical position and could not be visualized through the defect. After the removal of adhesions around the perimeter of the hernia, traction was applied to the omentum to reduce it into the abdominal cavity. The reduction of the omentum also led to the reduction of the colon and stomach into the abdominal cavity without additional effort, and the liver could be visualized in the right thoracic cavity through the diaphragmatic defect, along with an atrophic right lung. At this point, a subcostal incision was made connecting the initial two port sites, and a hand port was placed. The diaphragmatic defect was extended laterally (with care taken to avoid damaging the posterolateral branch of the phrenic nerve) to assist in the subsequent adhesiolysis. The liver was palpated, and gentle traction applied so that adhesions could be taken down. A single thoracoscopic port was placed (Fig. 4e) to allow a better visualization during this phase of the procedure. Once the liver was free of intrathoracic adhesions, it was gently pulled down and reduced to its anatomical position and the remaining hernial sac was resected. Repair of the diaphragmatic defect began from lateral to medial via an abdominal approach using interrupted Ethibond (Ethicon, Somerville, NJ) polyester sutures, but once the diaphragm could no longer be well approximated, a combined thoraco-abdominal approach was required. A 7-cm lateral incision was made by extending the thoracoscopic port site into the eighth intercostal space, and a segment of the eighth rib was resected. The liver was palpated and retracted through the abdominal hand port to allow for better exposure of the diaphragm, while the remaining approximation of the diaphragmatic rupture was completed via the thoracic approach. The repair was reinforced with a 10 × 15-cm Covidien ProGrip (Medtronic, Minneapolis, MN) synthetic mesh that was applied and tacked into place laparoscopically from the abdominal side (Fig. 5b). Operative time was 220 min and the total estimated blood loss was 100 mL.

**Fig. 6:**
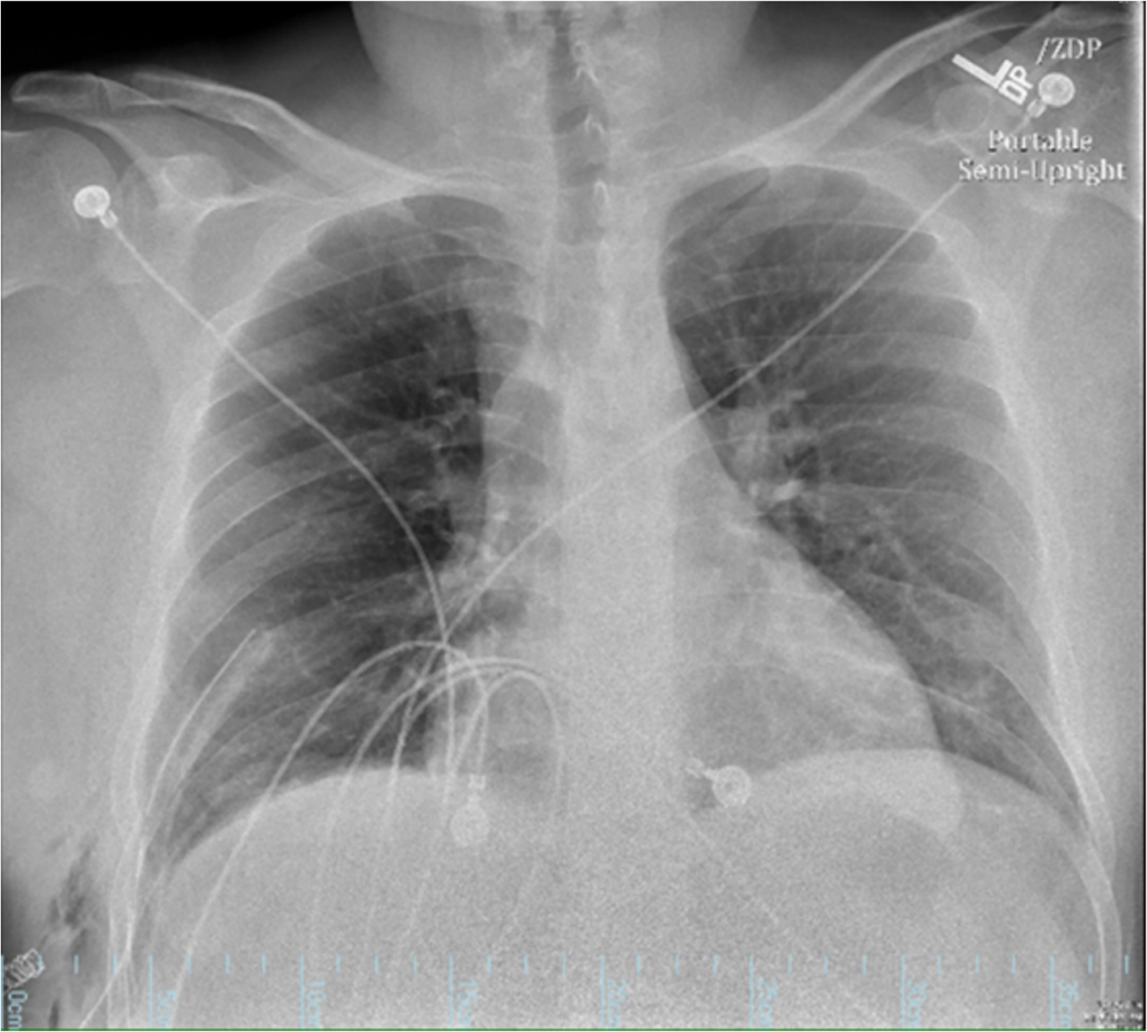
Post-operative chest radiograph showing good diaphragmatic repair and no remaining intrathoracic herniation

The patient had an uneventful post-operative course. Post-operative chest radiograph showed a good anatomical repair (Fig. 6). He was discharged on the eighth post-operative day on a regular diet. Six months after the procedure, the patient is doing well and shows no evidence of recurrence.

## Discussion

The diaphragm is a muscular structure which separates the high-pressure abdominal cavity from the negative pressure of the thoracic cavity. Traumatic diaphragmatic injury occurs in 0.8 to 8% of all thoraco-abdominal traumas. Seventy-five percent of TDIs are due to blunt trauma and 25% to penetrating [4].

When the abdomen is impacted by a strong enough blunt force, a sudden increase in intrabdominal pressure occurs. If this increase in pressure is greater than the tensile strength of the diaphragm, then the diaphragm ruptures, typically resulting in defects larger than those caused by penetrating injuries, which reflect the size of the penetrating agent [2, 4].

Only 20% of blunt traumatic diaphragmatic injuries occur on the right side [2, 3, 5–7]. The functional anatomy of the diaphragm is the reason behind both the rarity and the diagnostic difficulty of right-sided traumatic diaphragmatic injury. The close proximity of the liver to the inferior aspect of the right hemidiaphragm creates a physical barrier which protects the diaphragm from injury and acts as a cushion to attenuate the transmitted force from a blunt trauma to the abdomen [2, 4]. Furthermore, when right-sided diaphragmatic ruptures do occur, the dome of the liver can act as a seal of the diaphragmatic defect. This poses significant challenges to the early radiological visualization of the injury and, in association with a lack of immediate symptoms, can be falsely reassuring [6, 8].

When the diagnosis of diaphragmatic rupture is missed at the time of the inciting event, the pressure gradient between the abdominal and thoracic cavities causes a gradual migration of intrabdominal viscera into the thoracic cavity, resulting in delayed diaphragmatic hernia formation [9].

The clinical presentation of delayed blunt traumatic diaphragmatic hernias is variable and reflects the anatomical progression of each case. Patients may present weeks, months, or even many years after the initial trauma. When hernial orifices are smaller, creating narrow-necked hernia sacks, there is more risk of obstruction or strangulation of the hernial contents [10]. In many cases, the first presentation of a delayed diaphragmatic hernia may be due to symptoms of obstruction of herniated hollow viscera or strangulation and compromised blood supply to contents of the hernia, the latter often requiring urgent surgical intervention [2, 9]. Patients may also present with cardiopulmonary symptoms due to compression of intrathoracic structures by the hernial sac. In chronic hernias, abundant adhesion formation can further interfere with the normal function of both intrathoracic structures and the contents of the hernia sac [1]. In some cases, the anatomy of the hernia could allow relatively normal physiological function, and the diagnosis can be made completely incidentally on routine check-ups or while investigating other conditions [11]. The broad clinical and pathophysiological spectrum of delayed diaphragmatic hernias necessitate that each case be managed uniquely. Some cases, such as this one, may present in stable condition and can undergo an elective procedure, while some require much more urgent surgical intervention [3, 9]. A high index of clinical suspicion for delayed traumatic diaphragmatic hernia should be maintained for all patients with a history of trauma, but occasionally patients may present with a diaphragmatic hernia without a history of significant trauma [12]. Chest radiographs have a low sensitivity for detecting delayed diaphragmatic hernias, so even the slightest suspicion should be confirmed with thoracoabdominal CT as it is much more reliable for diagnosis of diaphragmatic hernias [2].

No matter what the presentation, all cases of delayed diaphragmatic hernia will need surgery to be corrected [1]. The specifics of the procedure however, much like the presentation, can vary greatly. If there is minimal herniation of abdominal viscera, a purely thoracic approach is often viable to repair the defect [6]. In some cases, an abdominal approach, either laparoscopically or via laparotomy, is sufficient. The chronicity of the hernia is also important to consider while planning for surgery as longstanding hernias can result in significant adhesion formation, further complicating the procedure. In these cases, excessive adhesions may necessitate a combined thoracoabdominal approach to satisfactorily lyse all adhesions, reduce the contents of the hernia to anatomical position, and repair the defect [12]. This further stresses the importance of early diagnosis in delayed traumatic diaphragmatic hernias.

## Conclusions

Management of delayed diaphragmatic hernias can be challenging, complex situations may require multidisciplinary coordination, but with meticulous planning and versatile surgical skills, a repair can be achieved resulting in good recovery and low risk of recurrence.

## Data Availability

All data generated or analyzed during this study are included in this manuscript.

## Abbreviations

CT: Computed tomography
ED: Emergency department
TDI: Traumatic diaphragmatic injury
